# Effects of Skin Blood Flow Fluctuations on Non-Invasive Glucose Measurement and a Feasible Blood Flow Control Method

**DOI:** 10.3390/s25041162

**Published:** 2025-02-14

**Authors:** Qing Ge, Tongshuai Han, Xueying Liu, Jiayu Chen, Wenbo Liu, Jin Liu, Kexin Xu

**Affiliations:** 1 State Key Laboratory of Precision Measurement Technology and Instruments, Tianjin University, Tianjin 300072, China; geqing@tju.edu.cn (Q.G.); hts2014@tju.edu.cn (T.H.); liuxveying@tju.edu.cn (X.L.); jiayu_chen@tju.edu.cn (J.C.); wenbo_liu@tju.edu.cn (W.L.); 2 Sunrise Technology Co., Ltd., Tianjin 300192, China

**Keywords:** near-infrared spectroscopy, non-invasive blood glucose measurement, blood flow pre-stimulation, blood flow background interference

## Abstract

In non-invasive blood glucose measurement (NBGM) based on near-infrared spectroscopy, fluctuations in blood flow represent a primary source of interference. This paper proposes a local blood flow pre-stimulation method in which the local skin is heated to dilate blood vessels and increase blood flow. This approach aims to mitigate the impact of environmental temperature variations, emotional fluctuations, and insulin secretion on blood flow, thereby enhancing the accuracy of glucose measurement. To evaluate the effectiveness of this method, a blood flow interference experiment was conducted to compare the stability of the measured spectra with and without blood flow pre-stimulation. The results demonstrated that the pre-stimulation method presents good anti-interference capabilities. Furthermore, 45 volunteers underwent oral glucose tolerance tests (OGTTs) as a part of the validation experiments. In these tests, the forearm skin blood flow of 24 volunteers was pre-stimulated using elevated temperature, while the skin of the remaining 21 subjects was maintained at a natural temperature level without stimulation. The results indicate that compared to the non-stimulated condition, the correlation between the optical signal at 1550 nm and blood glucose levels was significantly enhanced under the pre-stimulation condition. Furthermore, the root mean square error (RMSE) of the linear prediction model was reduced to just 0.92 mmol/L. In summary, this paper presents a feasible blood flow control strategy that effectively stabilizes internal blood flow, thereby improving the accuracy of NBGM.

## 1. Introduction

Regular glucose monitoring is indispensable for effective diabetes management. Currently, glucose monitoring is predominantly carried out through invasive methods, such as fingerstick tests and subcutaneous sensors. However, these approaches can cause discomfort and increase the risk of infection, and some sensor devices require frequent calibration. Wearable blood glucose meters, based on optical technology, offer the advantage of non-invasiveness and continuous monitoring, which are of profound significance for the prevention and treatment of diabetes. Since the 1980s, researchers have attempted to apply various methods for non-invasive blood glucose detection. Optical detection techniques such as optical polarimetry [1,2,3,4], infrared spectroscopy [5,6,7], optical coherence tomography (OCT) [8,9], Raman spectroscopy [10,11,12], and photoacoustic spectroscopy [13,14,15] have developed rapidly. Among these methods, near-infrared spectroscopy has attracted significant attention due to its ability of near-infrared radiation to penetrate soft tissues. Moreover, with the maturation of near-infrared devices, it has become one of the most promising non-invasive detection methods [16].

The main difficulties faced by NBGM using NIR spectroscopy at present are as follows: the glucose signal in the human body is relatively weak, and it is easily disturbed by a variety of measurement conditions. These disturbances mainly include changes in external measurement conditions and internal measurement conditions. Among them, the change in external measurement conditions is related to the change in the instrument (such as the drift of the light source, the temperature drift of the detector, and so on) and the change in the human–machine interface (such as the change in pressure at the interface, the change in position, the change in the angle of light incidence, and so on); the change in internal measurement conditions mainly refers to the interference of human physiological background, such as sweating, blood flow changes, tissue water content changes, and so on [17,18].

Our research team has rich experience in controlling measurement conditions. For example, Han et al. developed a sensor with a high signal–noise ratio (SNR) and multiple source–detector separations (SDSs) configuration, which effectively improved the stability of optical signals during human measurement [19]. In addition, Han et al. also proposed a posture reset method, which can restore the skin morphology to the initial measurement state every time. The oral glucose tolerance test (OGTT) experiment using these methods proposed by Han et al. has successfully detected the optical signal related to blood glucose changes at a single wavelength of 1550 nm [19]. Compared with external measurement conditions, internal measurement conditions, that is, the change in human physiological background, are difficult to control. If they are not well controlled, the spectral noise caused by physiological factors such as changes in blood flow will be introduced into the optical signal, resulting in the failure to observe the optical signal changing with blood glucose directly.

After extensive experiments, we observed that under the conditions of elevated skin temperature (36–42 °C), compared with the natural state of skin temperature (30.5–32 °C), it is easier to directly detect optical signals related to blood glucose changes at a single wavelength [19,20]. Based on this observation, we speculate that this phenomenon may result from differences in skin blood flow patterns between the two temperature conditions. Specifically, heating the skin might alter the local blood flow dynamics in the measured area, maintaining relative stability throughout the OGTT process, and thereby ensuring consistent internal measurement conditions. Consequently, we consider stable blood flow a crucial prerequisite for the direct measurement of blood glucose signals at a single wavelength.

In daily life, variations in blood flow are typically induced by multiple factors such as ambient temperature, emotional fluctuations, and blood pressure changes, and are also influenced by postprandial physiological responses like alterations in blood glucose levels and insulin secretion [21,22,23,24,25,26]. These factors lead to changes in blood volume and vascular status, affecting the propagation path and absorption characteristics of the detection light, which in turn modifies the background conditions for blood glucose measurements [27,28]. This complexity makes it challenging to extract clear blood glucose signals from the background noise.

To mitigate the impact of blood flow variations on spectral analysis, many studies have attempted to employ mathematical correction methods [27,29,30,31]. For instance, by collecting calibration datasets in advance and building models to eliminate or compensate for the interference caused by blood flow changes. However, our practical experience shows that these methods are not always effective because blood flow variations often occur rapidly after eating, coinciding with insulin secretion, which can cause the model to mistakenly identify these dynamic changes as blood glucose level alterations. Additionally, there exists individual variability in insulin secretion, including cases of deficiency or delayed secretion [32,33], further increasing the uncertainty in the relationship between blood flow and blood glucose. Therefore, considering blood flow changes as an interference rather than a usable signal is more appropriate.

It is worth noting that apart from blood flow and blood glucose, many other factors can also cause changes in tissue scattering properties, and the spectral features resulting from these changes lack specificity. Under such circumstances, relying solely on mathematical means may not effectively distinguish between different sources of contribution. In summary, over-reliance on mathematical methods to address complex interference issues in human measurement carries certain risks.

This paper proposes a method for reducing interference caused by factors such as insulin secretion and emotional fluctuations through preheating to stimulate blood flow in the measurement area. When human skin is subjected to heating stimuli, endothelial cells produce nitric oxide (NO), a potent vasodilator that activates the cGMP (cyclic guanosine monophosphate) pathway in smooth muscle, leading to the relaxation of smooth muscle fibers and subsequent vasodilation [34,35]. After food intake, insulin secretion increases, and insulin, by binding to receptors on vascular endothelial cells, further promotes the production of NO, resulting in vasodilation [36]. Notably, the vasodilatory effect of NO is regulated by biochemical pathways, physical limitations of vascular smooth muscle, and biological feedback mechanisms [37,38], preventing uncontrolled vessel expansion. Instead, vessels expand to a certain extent and then the effect ceases. Utilizing this characteristic, if the temperature of local skin is maintained at a higher level using an external heating module beforehand, the action of NO can increase blood flow to a relatively high state. At this point, further blood flow changes induced by factors such as insulin secretion will be diminished, thereby reducing their interference. Forst and others also found that when skin temperature was increased from 37 °C to 44 °C, there was a significant attenuation of blood flow changes after glucose solution intake in subjects [39].

This paper designed experiments to obtain the spectral changes caused by changes in arm posture, and we think that these are mainly caused by changes in blood flow. Therefore, we use this spectrum as the spectrum of the blood and compare it with the glucose spectrum. The results show that the two are very similar, which further emphasizes the necessity of controlling blood flow. In addition, we presented 45 experimental data cases of OGTT to substantiate the efficacy of the pre-stimulation (preheating skin) approach. Participants who underwent pre-stimulation formed the experimental group, whereas those who did not were designated as the control group. These experiments provided statistical confirmation of the pre-stimulation method’s effectiveness.

## 2. Methods

### 2.1. Glucose Measurement Based on Diffuse Reflectance Spectroscopy

**Figure 1:**
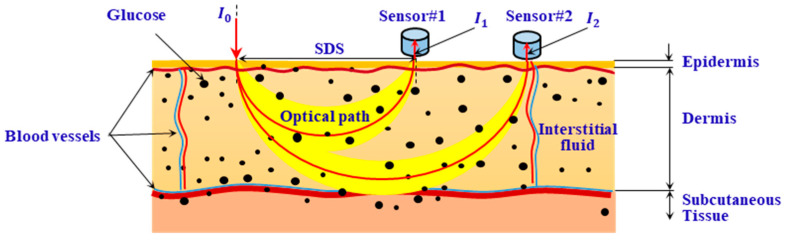
Illustration of glucose measurement based on diffuse reflectance spectroscopy.

Figure 1 shows a schematic diagram of the measurement using diffuse reflectance spectroscopy. In Figure 1, $I_{0}$ represents the intensity of the incident light, and $I_{1}$ and $I_{2}$ represent the intensity of the diffusely reflected light at two SDSs ($\rho_{1}$ and $\rho_{2}$) away from the incident point. The absorbances $A_{1}$ and $A_{2}$ at $\rho_{1}$ and $\rho_{2}$ can be calculated as follows:

(1) $$A_{1}=-ln\left(\frac{I_{1}}{I_{0}}\right),A_{2}=-ln\left(\frac{I_{2}}{I_{0}}\right),$$

To cancel the common-mode interference caused by instrument drift and unstable measurement conditions during in vivo measurements, differential data processing is performed. The measurement signal is expressed by differential absorbance $A_{D}$:

(2) $$A_{D}=A_{2}-A_{1}=ln\left(\frac{I_{1}}{I_{2}}\right),$$

### 2.2. NIR Diffuse Reflectance Spectra Caused by Blood Flow Change

This paper utilizes light in the waveband of 1000–1700 nm to detect changes in glucose concentration within the interstitial fluid of the dermal layer of the skin. Changes in glucose concentration led to alterations in tissue absorption and scattering properties. Additionally, the main components of blood—hemoglobin and water—also affect light absorption in the 1000–1700 nm wavelength range. Furthermore, variations in the number of red blood cells influence the scattering coefficients of tissues [40].

**Figure 2:**
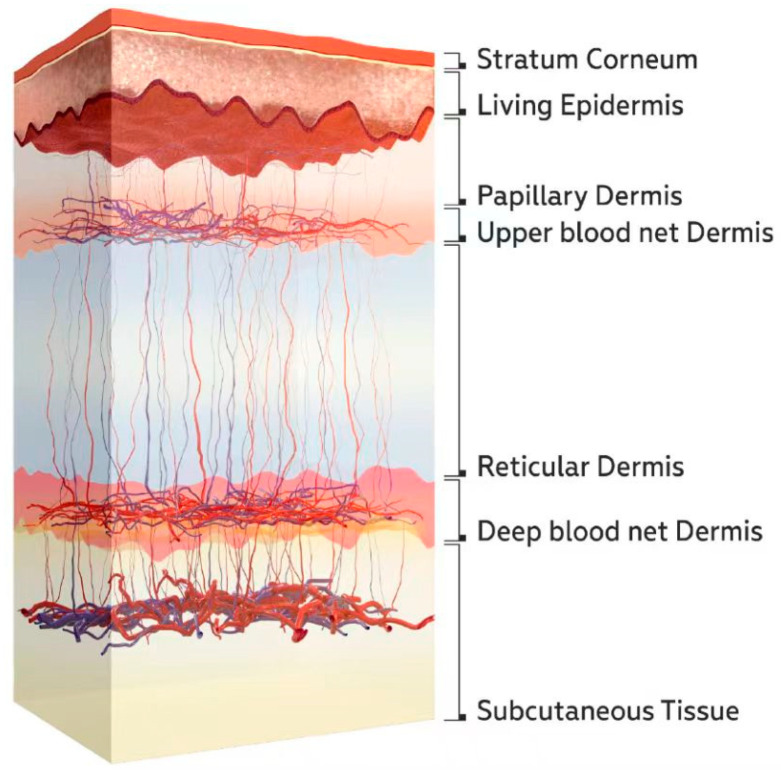
Schematic diagram of the skin structure [40].

A schematic diagram of the skin structure is shown in Figure 2. Blood vessels are primarily concentrated in the upper and deep blood net dermis. There are fewer blood vessels in the reticular dermis layer between these two layers. Therefore, changes in skin blood flow mainly affect the optical parameters of the upper and deep blood net dermis.

#### 2.2.1. Monte Carlo Simulation of the Spectrum Caused by Skin Blood Flow Variation

Monte Carlo (MC) simulation was used to estimate the spectra of skin blood flow variations. The following section describes how the absorption and scattering coefficients of each skin layer are obtained, as well as how these values change after blood flow variations.

Equations (3)–(5) are used to describe the calculation method for the absorption coefficients of various skin layers in Figure 2.

The epidermis is further subdivided into the stratum corneum and the living epidermis, whose absorption coefficients are calculated using Equations (3) and (4), respectively. This calculation method references the research findings of Meglinski et al. [41].

(3) $$\mu_{a}^{S}\left(\lambda \right)=\left(0.1-8.3\times 10^{-4}\times \lambda \right)+0.125\times \mu_{a}^{Other}\left(\lambda \right),$$

(4) $$\mu_{a}^{L}\left(\lambda \right)=\left(0.5\times 10^{10}\times \lambda^{-3.33}\right)(1-C_{H_{2}O})+C_{H_{2}O}\mu_{a}^{H_{2}O}\left(\lambda \right),$$

**Figure 3:**
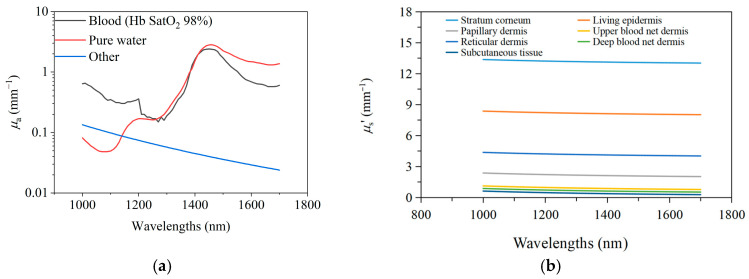
The optical parameters. (**a**). The $\mu_{a}^{H_{2}O}\left(\lambda \right)$, $\mu_{a}^{B}\left(\lambda \right)$, and $\mu_{a}^{Other}\left(\lambda \right)$ in 1000~1700 nm wavelength band; (**b**). the reduced scattering coefficients $\mu_{s}^{\prime }$ of each skin layer.

Here, $\mu_{a}^{S}\left(\lambda \right)$ and $\mu_{a}^{L}\left(\lambda \right)$ are the absorption coefficients of the stratum corneum and living epidermis, respectively. $\mu_{a}^{H_{2}O}\left(\lambda \right)$ is the absorption coefficient of pure water; $\mu_{a}^{B}\left(\lambda \right)$ is the absorption coefficient of blood; and $\mu_{a}^{Other}\left(\lambda \right)$ is the absorption coefficient of components in the tissue other than water and blood. Figure 3a shows the typical values of $\mu_{a}^{H_{2}O}\left(\lambda \right)$, $\mu_{a}^{B}\left(\lambda \right)$, and $\mu_{a}^{Other}\left(\lambda \right)$. We assume that there is no blood in the stratum corneum and the viable epidermis.

For the calculation of absorption coefficients of the dermis (including the upper blood net dermis, papillary dermis, reticular dermis, and deep blood net dermis) and the subcutaneous tissue layer, we have adopted Equation (5). Since these five layers have a relatively complex structure and contain varying amounts of blood and water, three variables are introduced in Equation (5). These variables represent the volume fraction of blood, water in interstitial fluid, and other components in each layer, denoted as $C_{B}$, $C_{H_{2}O}$, and $C_{Other}$. Here, $C_{Other}=1-C_{H_{2}O}-C_{B}$. The typical values of $C_{B}$ and $C_{H_{2}O}$ are listed in Table 1 [41]. This method of calculating the absorption coefficient of the entire tissue layer by integrating the contributions of various components to absorption is currently a commonly used approach.

(5) $$\mu_{a}^{DS}\left(\lambda \right)=C_{B}\mu_{a}^{B}\left(\lambda \right)+C_{H_{2}O}\mu_{a}^{H_{2}O}\left(\lambda \right)+(1-C_{H_{2}O}-C_{B})\mu_{a}^{Other}\left(\lambda \right),$$

where $\mu_{a}^{DS}\left(\lambda \right)$ represents the absorption coefficient of a layer in the dermis or the subdermal tissue layer.

The reduced scattering coefficients $\mu_{s}^{\prime }$ of each skin layer are obtained from references [20,41,42], as shown in Figure 3b.

Since the active regions of blood flow generally appear in the upper blood net dermis and deep blood net dermis, this paper only considers the changes in skin spectra due to variations in blood volume in these two layers. Consequently, we utilized Equations (6)–(9) to calculate the changes in absorption and scattering coefficients of the upper blood net and deep blood net due to variations in blood volume fraction $C_{B}$ while keeping the absorption and scattering coefficients of all the other layers constant during this process.

According to Equation (5), the changes in the absorption coefficients $∆\mu_{a}^{DS}$, for the upper blood net dermis and the deep blood net dermis, can be calculated as follows:

(6) $$∆\mu_{a}^{DS}={∆C}_{B}\cdot \mu_{a}^{B}+∆C_{Other}\cdot \mu_{a}^{Other},$$

where $∆C_{Other}=k\cdot ∆C_{B}$. We believe that the change in $C_{B}$ will lead to corresponding changes in $C_{Other}$. Specifically, when the volume fraction of blood increases, the total volume fraction of other components will decrease accordingly. We assume that the proportionality between ${∆C}_{B}$ and $∆C_{Other}$ is constant and k≈−1. Additionally, we assume that the water content of interstitial fluid $C_{H_{2}O}$ remains constant during this process.

The changes in $\mu_{s}^{\prime }$ caused by blood variations can be derived based on the Mie scattering theory [7,9]. The $\mu_{s}^{\prime }$ of the dermis can be expressed using Equation (7), and its change can be expressed in Equation (9):

(7) $$\mu_{s}^{\prime }=3.28\pi r^{2}\rho_{s}{\left(\frac{2\pi r}{\lambda }\right)}^{0.37}{\left(\frac{n_{s}}{n_{0}}-1\right)}^{2.09},$$

(8) $$\frac{∆\rho_{s}}{\rho_{s}}=\frac{∆C_{B}}{C_{B}},$$

(9) $$∆\mu_{s}^{\prime }=3.28\pi r^{2}{\left(\frac{2\pi r}{\lambda }\right)}^{0.37}{\left(\frac{n_{s}}{n_{0}}-1\right)}^{2.09}{\cdot ∆\rho }_{s}=\mu_{s}^{\prime }\frac{{∆\rho }_{s}}{\rho_{s}}=\mu_{s}^{\prime }\frac{∆C_{B}}{C_{B}},$$

where r, $\rho_{s}$, and $n_{s}$, is the radius, density, and refractive index of scattering particles, respectively, and $n_{0}$ is the refractive index of the solvent. We assume that when the $C_{B}$ changes, the values of r, $n_{0}$, and $n_{s}$ remain constant, while only $\rho_{s}$ varies. Moreover, we assume that the relative rates of change of $\rho_{s}$ and $C_{B}$ are the same, as shown in Equation (8). Equation (9) provides the differential of $\mu_{s}^{\prime }$ when $\rho_{s}$ changes, and it can further be seen that the $∆\mu_{s}^{\prime }$ can be calculated by using $\mu_{s}^{\prime }$ and $∆C_{B}/C_{B}$.

Table 1 shows the thickness of each skin layer, the blood and water contents, and the assumed variation in blood content [41], as well as the corresponding changes in the scattering coefficients ($∆\mu_{s}^{\prime }/\mu_{s}^{\prime }$) of the upper blood net dermis and the deep blood net dermis.

After calculating the changes in the absorption and scattering coefficients caused by blood variation, we input these values into the Monte Carlo (MC) simulation program to simulate the diffuse reflectance spectra of the skin before and after the blood change. By subtracting the simulated results, we obtain the theoretical blood spectrum.

The simulation program used is Monte Carlo eXtreme (MCX), version 1.9.7 (v2022.10, Heroic Hexagon), proposed by Shen et al. [43]. The simulation models a skin–probe interface with glass having a refractive index of *n* = 1.4 and a thickness of 0.2 mm. The number of incident photons is set to 10^10^. The SDS settings for the detectors are calibrated at 0.9 mm, 1.25 mm, 1.7 mm, 2.0 mm, and 2.3 mm, respectively. The MC simulation employs six distinct wavelengths, centered at 1050 nm, 1219 nm, 1314 nm, 1380 nm, 1550 nm, and 1609 nm, each accompanied by a bandwidth of 3 dB. The SDSs of detectors, as well as the central wavelengths and 3 dB bandwidths of six light sources, align with the specifications outlined in Section 2.3 of the instrument configuration.

#### 2.2.2. Experiment Measuring the Spectrum of Skin Blood Variation

This paper carefully designs a human experiment to obtain the spectrum generated by changes in blood flow in the skin of the human arm. Geraldine and others revealed an important phenomenon in relevant experiments: when the subject maintains a sitting posture, if the relative height between the arm and the heart changes, the blood content in the skin will also correspondingly change [44]. Based on this phenomenon, we have designed an experiment specifically for measuring the blood spectrum.

**Figure 4:**
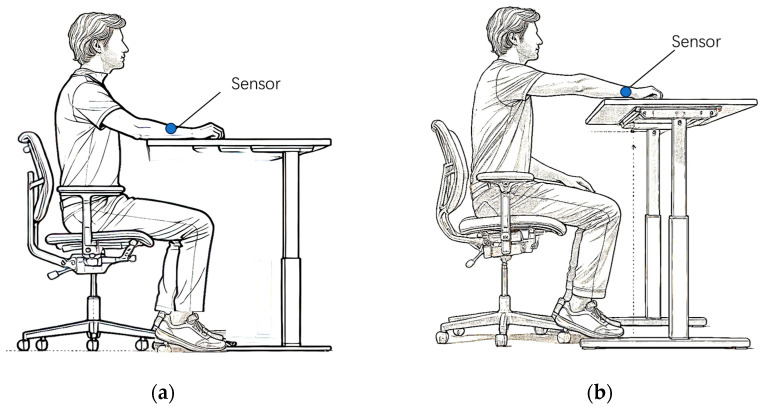
Schematic diagram for measuring blood spectrum in human forearm skin. (**a**). The forearm is placed at the height level of the heart. (**b**). The forearm is placed higher than the heart.

The subjects fasted for 12 h and rested in a quiet environment for at least 1 h before the experiment. A sensor was affixed to the subject’s arm. First, the subject was required to place the forearm stably on a lifting table. Then, by precisely adjusting the height of the lifting table, the subject’s forearm was brought as close as possible to the horizontal height of the heart, as shown in Figure 4a. After the subject’s forearm had been stable at this position for 4 min, skin spectra were collected and recorded as $A_{D0}$. Next, we kept the subject’s forearm in the same position on the table, and within a relatively short time interval (10 s), raised the lifting table by 20 cm, as shown in Figure 4b. After completing this operation, we collected the skin spectrum and recorded it as $A_{D1}$. The spectrum caused by blood changes was denoted as $A_{D,blood}$, which is calculated by the equation:

(10) $$A_{D,blood}=A_{D0}-A_{D1}$$

To ensure the reliability of the data, the above operations need to be repeated 3 times. For each subject, three blood spectra were obtained through the three operations, and the average of these three spectra was taken as the final blood spectrum. The entire measurement process takes a short time, approximately 12 min under stable ambient conditions (25 ± 0.2 °C, 55–60% humidity), with the subjects maintaining a fixed posture throughout in order to eliminate the potential impact of these factors on the experimental data.

### 2.3. The Blood Flow Pre-Stimulation Method Using High-Temperature Heating

We propose a method that utilizes high-temperature heating to pre-stimulate blood flow. After such stimulation, the blood flow will be maintained at a higher level for a specific period of time. Specifically, the measurement area will be heated to a temperature 4–6 °C higher than the normal skin temperature, which can effectively promote the endothelial cells to produce nitric oxide (NO). The production of NO will induce vasodilation, thereby increasing blood flow. Through continuous and prolonged heating, blood flow can be kept stable for a certain period of time. When the blood flow is stimulated, the role of nitric oxide induced by insulin will no longer be significant. During OGTT, blood flow can remain in a relatively stable state.

To verify the effectiveness of this blood flow pre-stimulation method, we compared the stability of skin spectra under conditions with and without pre-stimulating blood flow when external disturbances were applied to stimulate skin blood flow. External disturbances were applied to the skin near the measurement area and heated to affect its local blood flow, thereby indirectly influencing the blood flow in the measurement area.

**Figure 5:**
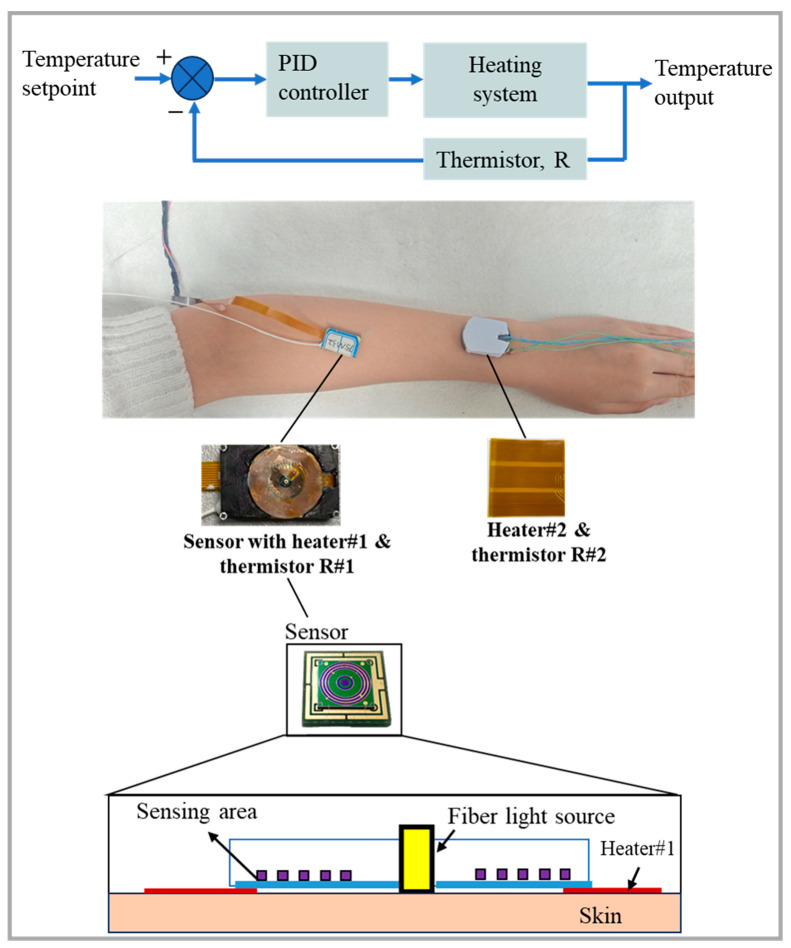
Measurement schematic diagram of the wearable sensor with a temperature control module (Heater #2 serves to elevate the temperature of the skin proximate to the measurement zone).

The temperature control module consists of a heater (a resistive wire wrapped in polyester) and a temperature feedback device. The heater acts directly on the skin and uses a thermistor to monitor the skin temperature, providing feedback for the PID control of the heater. The diffuse reflectance spectrum acquisition system includes a light source, an optical switch, five detectors, and a data acquisition/processing unit. The light source is composed of six super-luminescent diodes (SLDs, InPhenix, Livermore, CA, USA) with central wavelengths of 1050 nm, 1219 nm, 1314 nm, 1380 nm, 1550 nm, and 1609 nm, and 3 dB bandwidths of 51 nm, 32 nm, 36 nm, 58 nm, 52 nm, and 57 nm, respectively. Each diode is switched by a 6 × 1 optical switch (FSW1×1-SM-NL, CETC No.34, Guilin, China) and transmitted via optical fiber. The detectors are five independent, ring-shaped InGaAs photodiodes (U-Science Co., Ltd., Mukou-city, Kyoto, Japan) with the corresponding SDS of 0.9 mm, 1.25 mm, 1.7 mm, 2.0 mm, and 2.3 mm, respectively, and each ring has a width of 0.2 mm, as shown in Figure 5.

The experimental procedure is as follows: The sensor worn on the subject’s arm is used for skin spectra measurement and is equipped with Heater #1 and Thermistor #1 for heating and skin temperature monitoring, respectively. Heater #1 heats the skin and maintains the skin at a constant 36.5 °C to pre-stimulate blood flow. After heating for 30 min to 1 h, blood flow and spectral data stabilize (with differential absorbance $A_{D}$ fluctuations within 0.001 a.u. over 10 min). At this moment, an external disturbance is applied by activating Heater #2, which heats the adjacent skin to 39 °C, and the stability of the skin spectra in the measurement area is observed. In the control experiment, the skin temperature in the measurement area is maintained at 32.5 °C near the natural skin temperature without pre-stimulating blood flow. Heater #2 is then used to apply an external disturbance to stimulate blood flow in the adjacent skin, and changes in the skin spectra in the measurement area are observed. The ambient temperature is kept constant at 25 ± 0.2 °C throughout the experiment.

### 2.4. Human Validation Experiment Using OGTT

We conducted OGTTs to confirm that the method of pre-stimulating blood flow can effectively improve the accuracy of glucose measurement. The OGTTs were carried out under two conditions: pre-stimulating blood flow (experimental group) and no blood flow stimulation (control group). In a controlled environment meticulously maintained at a constant temperature of 25 ± 0.2 °C, our experimental procedures were carried out. Despite the standardized conditions, individual physiological differences led to a range of skin temperatures among the participants, which were approximately between 30.5 and 32 °C. Based on the research findings of Samah et al., we believe that when the skin temperature is controlled at 32.5 °C, which is an increase of only 0.5–2 °C from the baseline, the skin blood flow is almost unaffected [45]. Therefore, in the control group experiment, a temperature control module was used to maintain the measurement area at 32.5 °C to avoid the influence of skin temperature fluctuations on the spectra. The correlation coefficients between single-wavelength optical signals and blood glucose concentration references in the two situations were compared.

The detailed information of the volunteers is shown in Table 2. All the subjects were healthy individuals.

All the tests were performed between 8 a.m. and 12 p.m., requiring the participants to fast and abstain from drinking water after 10 p.m. the previous night. An initial phase was necessary prior to the OGTT experiment. In the experimental group, the initial phase involved pre-stimulating blood flow for about 30 min to 1 h. During this period, the participants wore sensors on their forearms and heated them to 36.5 °C while sitting quietly in a room and resting, with their arms free to move. After heating for 30 min, the participants kept their arms still to collect spectral data and observe its stability. Once the data stabilized within 10 min (with fluctuations in differential absorbance $A_{D}$ less than 0.001 a.u.), the formal OGTT experiment began. In the control group, the initial phase usually requires less than 15 min, because the skin temperature was only controlled at 32.5 °C. After the initial phase, the participants consumed a sugar water solution containing 250 mL of liquid with 75 g of glucose. Subsequently, the participants maintained a fixed posture for continuous data collection for 1 to 3 h until their blood glucose levels dropped below 6 mmol/L. If the participants felt fatigued from maintaining one posture, they could pause data recording for 1 to 2 min, move around, and then resume the original posture and continue recording. The arm positioning used the double cross-laser positioning method proposed by Han et al. [19]. The indoor humidity was suitable, at about 55–60%, and was not controlled. Two portable glucometers (GT-1820, Arkray, Japan) were used to measure fingertip blood glucose every 5 min, and the average value was taken as the reference blood glucose value.

## 3. Results

### 3.1. Monte Carlo Simulation Results and Experimental Spectra Caused by Blood Flow Changes

**Figure 6:**
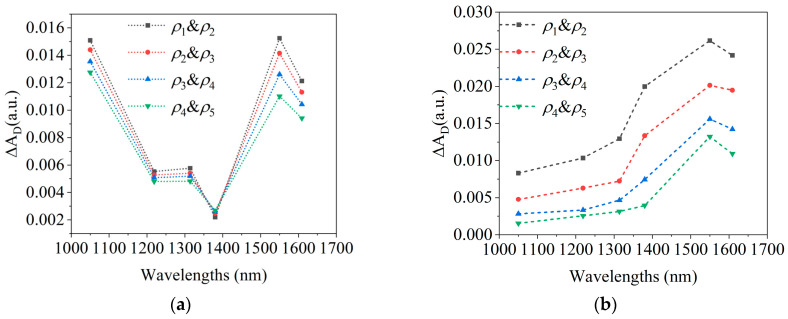
MC simulation results of the spectra caused by blood changes. (**a**). The effects of changes in $\mu_{a}$ on spectra; (**b**). the effects of changes in $\mu_{s}^{\prime }$ on spectra.

Figure 6a,b show the effects of changes in $\mu_{a}$ and $\mu_{s}^{\prime }$ on spectra, respectively, caused by blood variations as simulated by the MC model.

**Figure 7:**
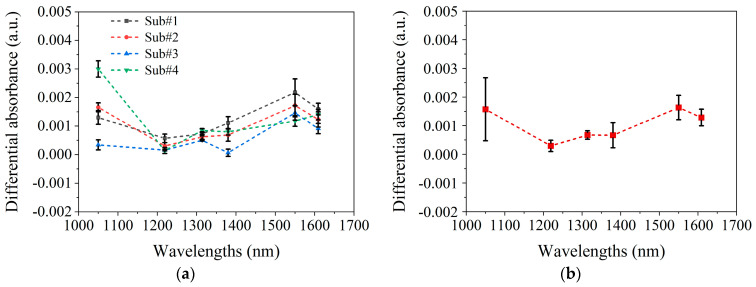
The experimental results of spectral changes caused by blood variations. (**a**). Mean and standard deviation of the three blood spectra $A_{D,blood}$ for four subjects, respectively. (**b**). Mean and standard deviation of four subjects’ final blood spectra $A_{D,blood}$.

Figure 7a presents the final blood spectra for each of the four subjects, which were averaged from three blood spectra acquired from three arm elevation operations. Figure 7b shows the average final blood spectra and variations among subjects.

In Figure 7b, the average spectrum of blood variations closely matches the MC-simulated blood absorption spectrum in Figure 6a. At 1050 nm and 1550 nm, ${∆A}_{D}$ is maximal and comparable in magnitude, with the greatest inter-subject variability being observed at 1050 nm. The MC simulation in Figure 6 shows that the differences between blood absorption and scattering spectra also primarily occur at 1050 nm, suggesting that variability arises from the differing contributions of the absorption and scattering effects among the subjects. For example, in subjects#1, #2, and #4, the larger $A_{D,blood}$ at 1050 nm indicates a greater influence of absorption, while subject#3’s spectrum suggests scattering dominates, as shown in Figure 7a. These differences likely result from variations in skin structure, measurement location, or physiological state.

**Figure 8:**
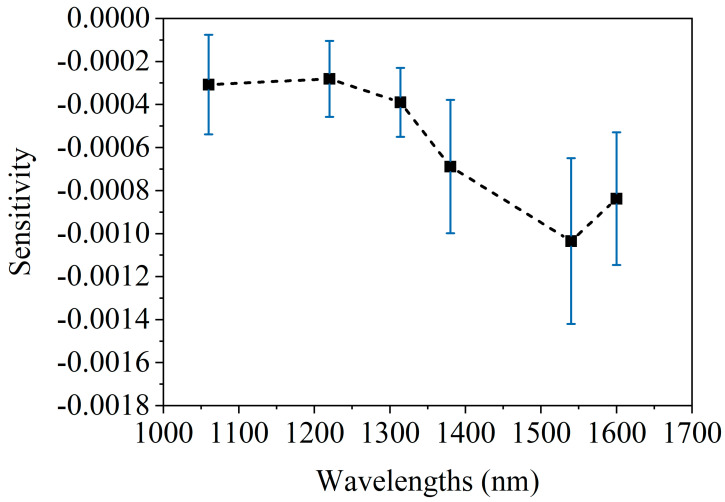
The average spectrum of $A_{D}$ caused by 1 mmol/L glucose concentration change, acquired on human forearm skins [20].

Figure 8 shows the spectrum of 1 mmol/L glucose change [20]. After comparing the blood glucose spectrum (Figure 8) with the blood spectrum (Figure 6 and Figure 7), it is found that the two have similar spectral characteristics, and both have peaks near 1550 nm. However, there are also some differences; that is, the blood spectrum has a peak at 1050 nm, whereas the blood glucose spectrum does not. These differences are small and not easily distinguishable. In addition, comparing Figure 7b and Figure 8 indicates that raising the arm 20 cm, altering blood flow, causes spectral changes equivalent to a 1–2 mmol/L glucose diminution. This further highlights the necessity of maintaining stable blood flow during the blood glucose measurement process.

### 3.2. Test Results Under Blood Flow Interference

**Figure 9:**
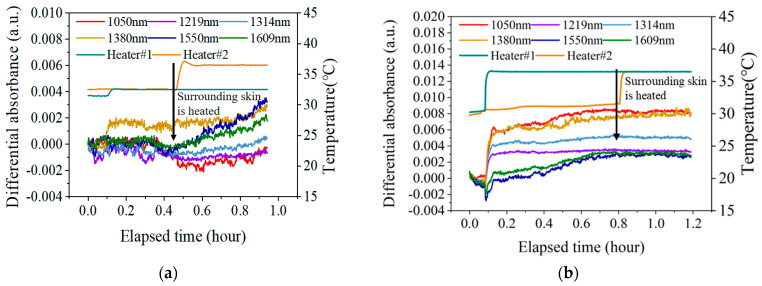
The changes in the measured $A_{D}$ after applying external blood flow disturbances by heating the skin near the measurement area. (**a**). The results without blood flow pre-stimulation; (**b**). the results with blood flow pre-stimulation.

Figure 9 shows the changes in the measured spectrum $A_{D}$ after applying external blood flow disturbances by heating the skin near the measurement area. Figure 9a shows the experimental results without blood flow pre-stimulation, and Figure 9b shows the experimental results with blood flow pre-stimulation.

As can be seen from Figure 9a, after heating the natural skin temperature to 32.5 °C, the $A_{D}$ at all six wavelengths remained stable, with fluctuations less than 0.001 a.u. over a period of 20 min. In addition, when the skin condition is stable, applying external blood flow disturbance (heating the adjacent skin area) will affect the blood flow state of the local skin, thereby causing spectral changes. Among them, the $A_{D}$ changes at 1550 nm and 1609 nm are more significant. At the wavelengths of 1050 nm, 1219 nm, and 1314 nm, where blood flow absorption is more obvious, most of the effects of blood flow are eliminated after differentiation.

As can be seen from Figure 9b, the blood flow pre-stimulation stage lasted about 40 min, and the spectra at all six wavelengths were stimulated to a certain level. Among them, the change of $A_{D}$ at 1550 nm was the biggest, and the spectrum increased by 0.004 a.u. However, after the external blood flow disturbance was applied, the $A_{D}$ at all six wavelengths remained stable and did not change further.

The above results indicate that the skin blood flow can effectively shield the further blood flow disturbance after pre-stimulation.

### 3.3. OGTT Results with and Without Blood Flow Pre-Stimulation

We conducted 24 OGTT cases with blood flow pre-stimulation and 21 OGTT cases without blood flow pre-stimulation. For each OGTT, we calculated the correlation coefficients (*R*) between the differential absorbance at 1550 nm ($A_{D}$) and glucose concentration ($C_{g}$). Fitting lines were constructed based on $A_{D}$ at 1550 nm v.s. $C_{g}$, and subsequently, the root mean square error (RMSE) between these fitting lines and the actual values of $C_{g}$ was determined. The statistical experiment results are presented in Table 3.

**Figure 10:**
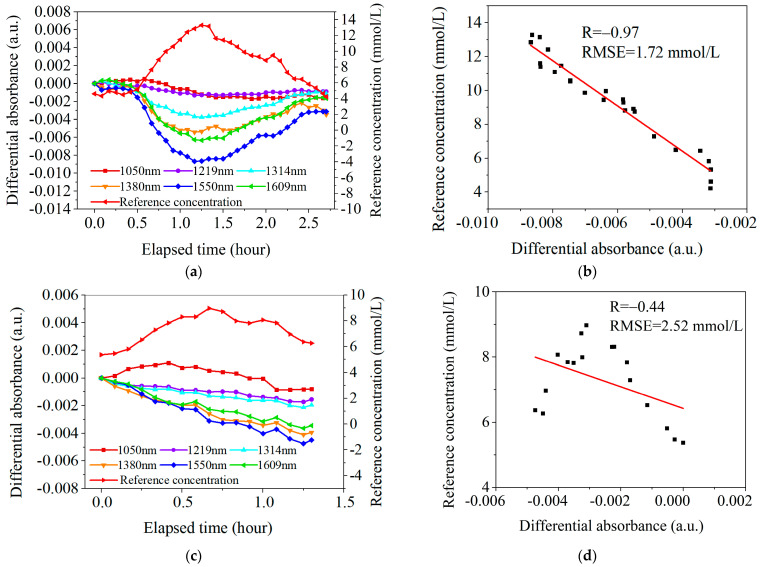
Two sets of typical OGTT data. (**a**). The results under the conditions of blood flow pre-stimulation (with skin temperature controlled at 36.5° C). (**b**). Linear fit between $A_{D}$ at 1550 nm and $C_{g}$ under the conditions of blood flow pre-stimulation. (**c**). The results without high-temperature pre-stimulation (with skin temperature controlled at 32.5 °C). (**d**). Linear fit between $A_{D}$ at 1550 nm and $C_{g}$ without high-temperature pre-stimulation.

According to the statistical analysis in Table 3, among the 24 OGTT experiments with blood flow pre-stimulation, 8 cases had a correlation coefficient *R* > 0.85, and only 1 case had a correlation coefficient *R* < 0.5, with a smaller RMSE of 0.91 mmol/L. Figure 10a,b show one classical case of the experimental results with the skin temperature controlled at 36.5 °C. In Figure 10b, the correlation coefficient *R* is 0.91 and the RMSE is 1.72 mmol/L.

In contrast, among the 21 OGTT experiments without blood flow pre-stimulation, only 1 case had a correlation coefficient *R* > 0.85, and as many as 14 cases had a correlation coefficient *R* < 0.5, with a larger RMSE of 6.12 mmol/L. Figure 10c,d show one classical case of the experimental results with the skin temperature controlled at 32.5 °C. In Figure 10d, the correlation coefficient *R* is 0.44 and the RMSE is 2.52 mmol/L.

## 4. Discussion

(1) The effectiveness of pre-stimulation with high-temperature heating in reducing blood flow interference and differences.

The measurement experiments of blood spectra have shown the differences in blood spectra among different individuals. In fact, even for the same subject, there are also slight differences among their three blood spectra. Given this, it is quite difficult to use a fixed blood spectrum for the blood glucose measurement model of a certain subject. At the same time, the OGTT experiment results show that the blood flow changes caused by insulin secretion during OGTT also present differences. In the control group (without blood flow pre-stimulation), among the 21 experiments, only 4 cases had ideal measurement results, with the correlation coefficient between $A_{D}$ and $C_{g}$ being higher than 0.7, which suggests that the blood flow state did not change significantly during the OGTT process; while the other 17 experiments had poor results, with the correlation coefficient being lower than 0.7, which is probably because the blood flow failed to maintain a stable state during the OGTT process. In the experimental group of 24 cases, 20 cases had good experimental results with a correlation coefficient higher than 0.7, and only 4 cases were lower than 0.7. The above results effectively confirm the effectiveness of the method with the pre-stimulation of high-temperature heating, which can actually reduce the blood flow interference caused by insulin secretion after eating, and stabilize the blood flow state during the OGTT period.

(2) The instability of the pre-stimulation blood flow method and potential improvement approaches.

The current pre-stimulation method adopted is not perfect and has certain limitations. To be specific, this method is currently only applicable for OGTT experiments lasting 1–3 h, and there is not enough evidence to prove that it can continue to maintain a stable blood flow state for a longer time. Relevant research points out that after long-term heating, blood vessels may display the “die away” phenomenon and then contract to a certain extent [46,47,48]. In this way, in the process of continuous blood glucose monitoring, the accuracy of glucose measurement will be affected. In future research work, we will focus on optimizing the heating strategy, including the time arrangement of heating, the adjustment of the heater area, etc. At the same time, we will actively explore other effective methods to change the blood flow, such as large-area radiation heating, etc. In addition, it is also necessary to conduct research on the reproducibility of the repeated application of blood flow pre-stimulation. If the degree of each blood flow pre-stimulation is different, then it is also necessary to consider implementing spectral correction under different blood flow levels so as to ensure the accuracy and reliability of the experimental results.

## 5. Conclusions

This research effectively addresses the challenge of skin blood flow variations in NBGM by introducing a novel mitigation strategy.

Through a combination of human trials and MC simulations, we have demonstrated that fluctuations in skin blood flow exhibit spectral features similar to those of blood glucose, thereby complicating differentiation using conventional mathematical methods. Our solution entails externally controlling local blood flow via targeted heating, which stabilizes blood flow prior to OGTT. By applying heat at approximately 36.5 °C for pre-stimulus durations ranging between 30 and 50 min, our approach significantly enhances the accuracy of glucose spectrum measurements by mitigating blood flow interference.

The results of our OGTT with blood flow pre-stimulation confirm that our method substantially improves the correlation coefficient between optical signals and blood glucose references. This suggests effective isolation from additional blood flow changes, including those induced by postprandial insulin effects.

In summary, our high-temperature pre-stimulation method represents a highly useful practice that boosts the measurement accuracy of blood glucose spectra. Moreover, it provides a valuable reference for advancing other optical-based human blood glucose monitoring technologies.

## Figures and Tables

**Table 1:** Parameter of seven skin layer settings for MC simulation.

k	Name	$\mathrm{The}\;\mathrm{Parameters}\;\mathrm{Used}\;\mathrm{to}\;\mathrm{Calculated}\;\mu_{a}$ $\mathrm{and}\;{∆\mu }_{a}$			$\frac{∆\mu_{s}^{\prime }}{\mu_{s}^{\prime }}$	Thickness (cm)
		$C_{B}$ (%)	$C_{H_{2}O}$ (%)	${∆C}_{B}$		
1	Stratum corneum	0	5	—	—	0.002
2	Living epidermis	0	20	—	—	0.008
3	Papillary dermis	4	50	—	—	0.02
4	Upper blood net dermis	30	60	+50%	+50%	0.01
5	Reticular dermis	4	70	—	—	0.16
6	Deep blood net dermis	10	70	+50%	+50%	0.012
7	Subcutaneous tissue	5	70	—	—	0.65

**Table 2:** The detailed information of the volunteers who participated in OGTT.

	Age (Cases)			Gender (Cases)		Baseline Blood Glucose Levels (mmol/L)	Total (Cases)
	20 < Age < 30	30 < Age < 50	50 < Age < 70	Male	Female		
For OGTT cases with blood flow pre-stimulation	10	10	4	13	11	5.0 ± 0.6	24
For OGTT cases without blood flow pre-stimulation	8	9	4	12	9	4.9 ± 0.7	21

**Table 3:** The correlation coefficients and RMSE for OGTT cases.

	*R* > 0.85	0.85 > *R* > 0.7	0.7 > *R* > 0.5	0.5 > *R*	Average RMSE (mmol/L)
For 24 OGTT cases with blood flow pre-stimulation	8	12	3	1	0.91
For 21 OGTT cases without blood flow pre-stimulation	1	3	3	14	6.12

## Data Availability

The original contributions presented in the study are included in the article; further inquiries can be directed to the corresponding authors.
